# COVID-19 response: mitigating negative impacts on other areas of health

**DOI:** 10.1136/bmjgh-2020-004110

**Published:** 2021-04-15

**Authors:** Tabitha A Hrynick, Santiago Ripoll Lorenzo, Simone E Carter

**Affiliations:** 1 Health and Nutrition Cluster, Institute of Development Studies, Brighton, UK; 2 Public Health Emergencies, UNICEF, Geneva, Switzerland

**Keywords:** health systems, health policy, public Health, COVID-19

## Abstract

‘Vertical’ responses focused primarily on preventing and containing COVID-19 have been implemented in countries around the world with negative consequences for other health services, people’s access to and use of them, and associated health outcomes, especially in low-income and middle-income countries (LMICs). ‘Lockdowns’ and restrictive measures, especially, have complicated service provision and access, and disrupted key supply chains. Such interventions, alongside more traditional public health measures, interact with baseline health, health system, and social and economic vulnerabilities in LMICs to compound negative impacts. This analysis, based on a rapid evidence assessment by the Social Science in Humanitarian Action Platform in mid-2020, highlights the drivers and evidence of these impacts, emphasises the additional vulnerabilities experienced by marginalised social groups, and provides insight for governments, agencies, organisations and communities to implement more proportionate, appropriate, comprehensive and socially just responses that address COVID-19 in the context of and alongside other disease burdens. In the short term, there is an urgent need to monitor and mitigate impacts of pandemic responses on health service provision, access and use, including through embedding COVID-19 response within integrated health systems approaches. These efforts should also feed into longer-term strategies to strengthen health systems, expand universal healthcare coverage and attend to the social determinants of health—commitments, both existing and new—which governments, donors and international agencies must make and be held accountable to. Crucially, affected communities must be empowered to play a central role in identifying health priorities, allocating resources, and designing and delivering services.

**Summary box**

- Pandemic responses, especially ‘lockdowns’ and associated measures (eg movement restrictions), make it difficult for people to provide and access health services, particularly in low-income and middle-income countries. Such disruptions have led to negative impacts on other areas of health, including non-communicable and infectious disease, sexual and reproductive, and newborn and children’s health, and gender-based violence.
- The range of pandemic-related supply and demand-side drivers of health impacts interact with baseline vulnerabilities (eg, lack of safe and adequate housing, water and healthcare) to further exacerbate these impacts.
- Social groups including women and children, the elderly, people living with disabilities, migrant workers, ethnic and religious minorities, refugees and displaced people, and people in conflict-affected settings are particularly vulnerable to broader health impacts.
- Integrated health system approaches which address COVID-19 alongside other disease burdens, as well as inclusive priority setting, resource allocation, programme design, monitoring and implementation, are essential for improving response and equity.

## Introduction

The COVID-19 pandemic has undermined capacity and efforts to address other health needs that are just as pressing as the virus itself, particularly in low-income and middle-income countries (LMICs). Ongoing pressure on governments to act on COVID-19 now to save ‘immediately identifiable lives’ rather than ‘statistical lives at risk’ has had and will continue to have both short-term and long-term negative consequences for health.^1^

This analysis, based on a longer paper produced by the Social Science in Humanitarian Action Platform,^2^ highlights the impacts of vertical responses to COVID-19 on health systems, services, and people’s access to and use of them in LMICs, where historic and ongoing underinvestments heighten vulnerability to a multiplicity of health threats. By ‘vertical’ responses, we refer to COVID-19 interventions focused primarily on preventing and containing the virus, without adequately ensuring other health services remain safely operational, accessible and used by people who need them. This paper provides insight for use by actors in government, agencies, organisations and local communities to design and implement more proportionate, appropriate, comprehensive and socially just responses that address COVID-19 without compromising other aspects of health, particularly in LMICs. It presents a more holistic picture of the broader health and health system impacts of the pandemic, and specifically, those resulting from responses to contain to it.

To identify broader impacts and their drivers under narrow time constraints, we conducted a rapid evidence assessment of both academic and grey literature in August 2020.^3^ Following a search in the academic database Web of Science, which yielded few relevant articles, we also relied on Google and Google Scholar searches, as well as extensive snowballing methods. Due to the rapidly changing situation surrounding the COVID-19 pandemic, we also identified emerging information through reliable news outlets and reporting from development, public health and humanitarian organisations and databases. This iterative strategy enabled us to include the most recent developments and to capture data not represented in formal research from a range of settings and across a range of health and health system issues to address our broad questions, and thus to provide a snapshot of some of the key ways in which pandemic responses have and are likely to continue having broader impacts on health and health systems. See online supplementary appendix for a more detailed description of our methods and limitations.

10.1136/bmjgh-2020-004110.supp1 Supplementary data

Ultimately, we argue there is a need to re-evaluate priorities and approaches in global health, moving past immediate action, both in the context of COVID-19 and other crises. ‘Whole of health’ approaches which account for the health trade-offs of COVID-19 response in the short term^1^ as well as address the health needs of diverse populations in the medium term to long term are crucial for just and effective health outcomes.

## Vertical responses to COVID-19 and drivers of broader health and health system impacts

### Health security and the legacy of vertical response

While the scale of response to COVID-19 is unprecedented, ‘disease exceptionalism’ and vertical strategies/responses are not new.^4 5^ For decades, through the frame of ‘health security’, the global health community has focused on targeted preparation for, identification of and response to infectious disease outbreaks when and where they emerge—usually in LMICs—not least, as suggested by a common critique, to stop them before they can threaten wealthier populations in the global north.^6^ This orientation may have come at the expense of more holistic and equity-based approaches to health. Alongside structural adjustment policies—which critics have also suggested have undermined many LMICs by, for instance, forcing them to cut spending on health and social programmes—the health securitisation regime may have contributed to chronically weak health systems which do not meet the needs of populations and, ironically, may actually result in increased risk of outbreaks of infectious disease in these settings.^7 8^ Furthermore, in the context of emergencies, a long-standing issue has been that ‘humanitarian response’ models often do not allow for investments that benefit people in the longer term.^9^ For instance, funding may be pledged and budgets made available to cover the trucking in of water to healthcare facilities during a health crisis, but not for investments in new clean water infrastructure.

### Responding to COVID-19 in LMICs

The scale of the COVID-19 pandemic and its impacts on wealthy countries and individuals have prompted claims that the virus ‘does not discriminate’. Indeed, the fact that it has significantly impacted global power centres and elites may very well have generated the enthusiasm to mobilise so robustly across the world. Such vigour is rarely on offer with regard to the persistent and still massive burdens of disease and health risks faced by LMICs, even when we are not in the midst of a global crisis.^10^ Powerful COVID-19-focused discourses and political pressures at the global and national levels have pushed these burdens further out of sight and mind, and emergency logics focused on understanding, preventing and containing COVID-19 have prevailed.

Early, targeted action against COVID-19 in LMICs was also spurred by real and legitimate fear of high morbidity and mortality caused by the virus, and the potential overwhelming of already weak health systems. Several months down the line, many of these countries, particularly on the African continent, have not experienced the catastrophic scenarios initially predicted.^11^ One model for Africa suggested it could expect 190 000 deaths to COVID-19 over the period of 1 year^12^—a small fraction of the over 2.7 million mostly preventable under-5 child deaths that occurred on the continent in 2018 alone.^13^ While such a contrast may make broader health and health system impacts of COVID-19 responses seem even more unjustified in these settings, the significant non-COVID-19 disease burdens in LMICs which have experienced more substantial direct impacts indicate a need to recognise, mitigate and address broader impacts in these settings as well.

### ‘Supply-side’ drivers of broader health impacts

Early moves by LMIC governments to prevent COVID-19 from spreading in their countries focused on emergency measures aimed at limiting in-person contact, similar to those initially enacted in high-imcome countries. Strategies across settings ranged from near complete ‘lockdowns’ to more partial interventions in daily life.^14^ The specific mix of these ‘non-pharmaceutical interventions’ (NPIs), their stringency and duration has ranged widely as governments have responded to COVID-19 transmission dynamics within particular political, social and economic contexts. Such NPIs have included

- Halting ‘non-essential’ work activity.
- Movement restrictions and strict border controls or closures.
- Suspension of public gatherings.
- Stopping or scaling down public and private transportation systems.
- Curfews and stay-at-home measures.
- Closure of schools and other public institutions.
- Suspension of non-essential health services.
- Modifications to health service delivery, including closure of brick-and-mortar facilities.

Whatever mix and intensity, many of these measures have been disruptive to the availability, accessibility and use of health services in LMICs, and the public health rationales for deploying them in these settings have been widely questioned.^15^ Restrictive measures also interact with already profound weaknesses in LMIC health systems to produce even worse health outcomes. Limited material resources, staff and space have been diverted to address COVID-19, further straining capacity to address the wide range of health needs of different LMIC populations. Table 1 presents examples of how these interventions and systemic limitations have had broader health system impacts, which in turn have consequences for other areas of health.

**Table 1:** Supply-side drivers of broader health system impacts related to vertical response

Driver	Additional explanation
Disruptions to medical supply chains	Global and local medical supply chains stopped or slowed activity as production, transport routes and border controls have been disrupted, resulting in shortages, delays and stockouts of essential health resources, including contraceptives,^38^ antimalarials,^39^ antiretrovirals^40^ and vaccines,^41^ with import-reliant countries being particularly vulnerable.
Transportation challenges	HCWs, informal carers and those requiring care may be unable to travel to deliver or receive it if transport systems are disrupted. A ban on motorcycle taxis in Uganda, for instance, relied on especially by poor and rural people, made it difficult for them to reach facilities.^42^ Several pregnant women died after attempting to walk to reach care.^43^
Facility closures	Both public and private health facilities have been intentionally closed, often due to lack of resources to continue operating safely (clean water, disinfectant, personal protective equipment and COVID-19 outbreaks among staff). In Karachi, Pakistan, 18% of child immunisation facilities closed during lockdown.^44^
Resource diversion	Closures or service reductions may also occur due to resources, including staff and facilities, being diverted/repurposed for COVID-19 response. A survey found that 20% of labs normally supporting TB and HIV diagnostics across 106 countries experienced severe disruption as they pivoted to focus on COVID-19.^45^ In Kenya, Iraq and Honduras, facilities and hospitals where pregnant women have traditionally given birth, if not shut down, were converted.^46^
Funding shortfalls	Governments and organisations reliant on aid to operate health services struggled as donors failed to provide funds, particularly at the grassroots.^47^ In Yemen, resource diversions and cuts to acute malnutrition services resulted in nearly 30 000 fewer children a month receiving life-saving care.^48^ Only 17% of 160 countries allocated additional funds to sustain non-communicable disease services.^49^
Adaptations to health service delivery	Service delivery has been modified to minimise COVID-19 infection risk, including via adoption of phone-based or digital platforms.^50^ In LMICs, access to mobile phones or other communications technology, credit, coverage, data, internet and skills—while increasing—remain limited among patients and HCWs.^51^ The need for strict infection prevention control for services requiring in-person care (eg, immunisations, medical testing and surgery) raises service delivery costs.
Failures of health communication	If people are unaware of whether and how services have changed, they may be unable to access needed care. In India, confusion about whether TB clinics were open (alongside transport restrictions) left patients with TB dangerously low on medicine. It took the government a month into lockdown to clarify that TB services should continue uninterrupted.^52^
Suspension of specific health services	Governments are encouraged to identify and sustain ‘essential’ services and suspend ‘non-essential’ ones, especially during acute COVID-19 outbreaks.^53^ However, even if services are declared essential, not everyone with power over access to them may agree. Women seeking sexual and reproductive services in Zimbabwe and Ghana have reported being stopped by security officials.^54^

HCW, healthcare worker; LMICs, low-income and middle-income countries; TB, tuberculosis.

#### Beyond lockdowns

As suggested in table 1, not all drivers of broader health system and health impacts derive from restrictive measures. Limited health system resources play a major role, as does their prioritisation. Equally important are the connections between negative health impacts and vertical approaches to other elements of response. It is widely acknowledged that an effective overall epidemic response requires a range of public health measures such as surveillance, contact tracing, testing, risk communication and community engagement. All of these are key pillars of epidemic response^16^—and yet, they may have blind spots for other areas of health if vertical approaches to them are taken. While it is important that medical testing for COVID-19 is available and functioning amidst an outbreak, this should not be done at the expense of testing for other diseases, such as tuberculosis (TB), HIV or malaria which do not go away in the face of COVID-19, and still cause considerable morbidity and mortality in many LMICs.^17^ Risk communication and community engagement focused solely on COVID-19 risks leaving people without crucial information on how to protect themselves from other still-present health risks (although COVID-19 preventive measures may also protect people from a range of other infectious conditions), and importantly, how they can seek care for other health conditions in rapidly shifting health system landscapes.

### Demand-side drivers of broader health system and health impacts

While it is hard to disentangle exactly what keeps people from accessing health services in any given context without detailed research, significant drops in use in some settings have been clear. In Kinshasa (Democratic Republic of Congo), for instance, researchers observed a nearly 40% drop in use of diabetes services from March to June of 2020.^18^ Although the impeding supply-side factors described earlier may contribute to low use, demand-side dynamics, including fear—and not only of the virus—social circumstances, loss of income and difficulty adhering to treatment also play a role. Table 2 includes additional explanation of these factors.

**Table 2:** Demand-side drivers of broader health system and health impacts

Driver	Additional explanation
Fear of infection	Individuals needing care, caregivers and HCWs may reasonably fear contracting COVID-19 at or in transit to health facilities or transmitting it to loved ones. Without adequate resources to protect themselves (personal protective equipment and clean water), HCWs may also refuse to work; in Nigeria, there were reports of HCWs refusing to handle TB testing samples because of fear they may be COVID-19.^55^
Fear of quarantine or isolation	Qualitative evidence suggests the consequences of being found to have COVID-19 may be perceived to be worse than not receiving care for it or other conditions. Quarantine and isolation may mean separation from security, income and family, including others needing care. In Uganda, some people did not seek medical care from hospitals, fearing being put into quarantine if found to have COVID-19.^56^
Fear of punishment or violence	Fear of harassment, violence, fines or imprisonment for disobeying restrictive measures may impact health seeking or provision. After the brutal beating of a driver transporting a pregnant woman to hospital after curfew in Kenya, it became difficult for women to find transport.^46^ Enforcers may also use the pandemic as pretext to harass already vulnerable LGBTQI people or sex workers, making it difficult or dangerous for them to travel to services.^57 58^
Increased caring responsibilities	Carers—mainly women—may be forced to leave their jobs (if they have not already lost them) to provide care for children and elders in the wake of school, nursery and support service closures. They may also be reluctant or unable to leave them home, or bring them along for fear of exposing them to COVID-19 while attempting to access services for themselves.^59^
Loss of income	Income losses due to unemployment may make it harder for people to travel to, or to pay for health services for themselves or loved ones. Evidence from the Democratic Republic of Congo suggests recent falls in family planning service use are more attributable to lack of money than fear of contracting COVID-19.^60^
Stigma	People with stigmatising conditions such as HIV may hesitate to access care through new pathways for fear of having their status revealed. Mistrust of digital platforms or inability to engage with a familiar doctor may discourage care-seeking. HCWs are also vulnerable to stigma, if perceived as a source of infection. Resulting abuse adds to immense psychological stress, intense work pressure and fear of infection.^61 62^
Difficulty adhering to treatment	Uncomfortable side effects can make it difficult for patients to take drugs for certain conditions (eg, TB and HIV) without support. Lack of food can increase this difficulty as it can exacerbate side effects like vomiting, which also diminishes drug effectiveness. Increased food prices and loss of income has made it difficult for LGBT+ people living with HIV in Uganda to buy food—the fever, headaches and weakness the drugs cause on an empty stomach make it difficult to sustain treatment.^57^

HCW, healthcare worker; LGBT+, lesbian, gay, bisexual and transgender/transsexual; LGBTQI, lesbian, gay, bisexual, transgender, queer and intersex; TB, tuberculosis.

### Interacting with and exacerbating baseline vulnerabilities

**Figure 1:**
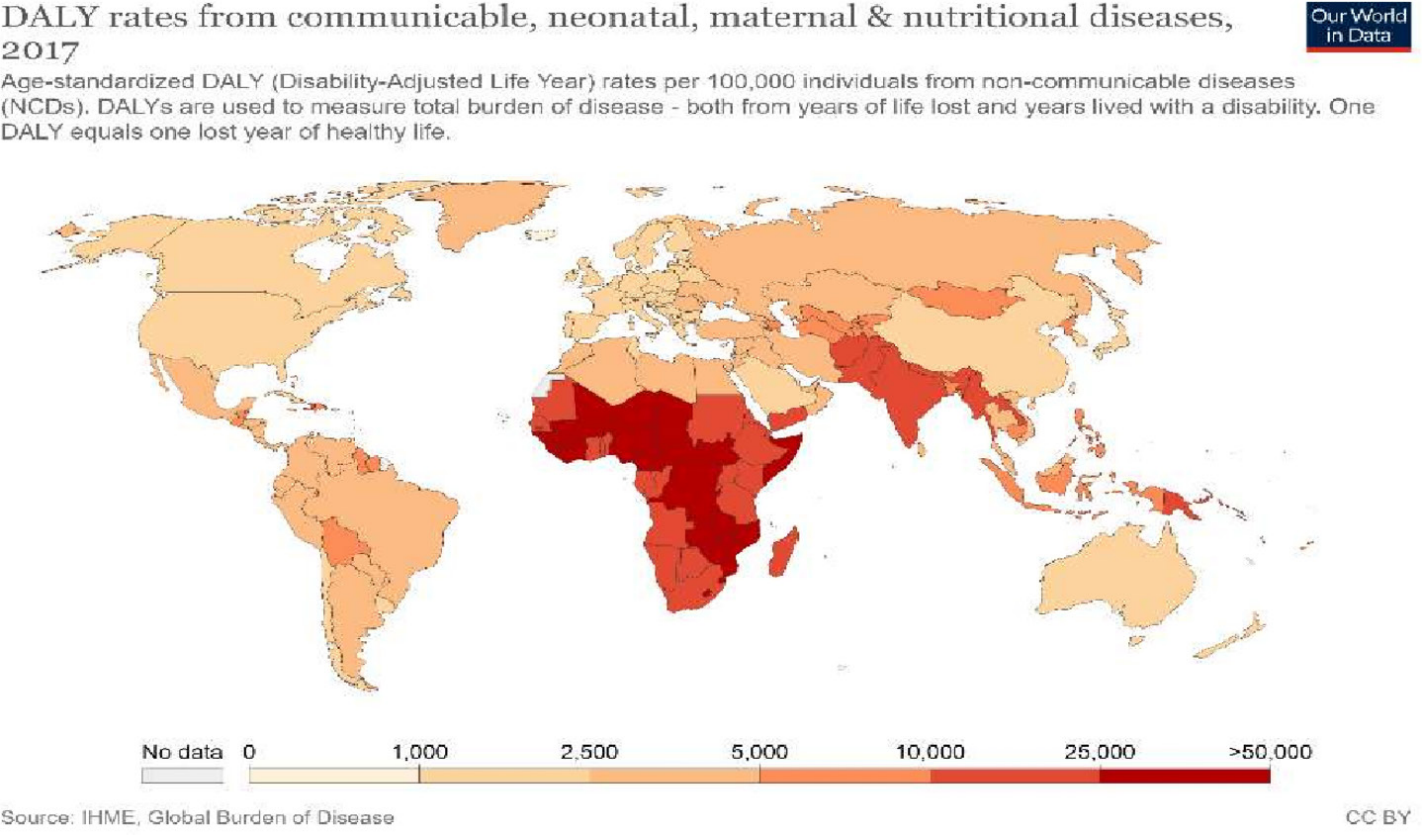
DALY rates from a range of disease and health issues. Source: Our world in data (2017), *DALY rates from a range of diseases and health issues* (https://ourworldindata.org/grapher/burden-of-disease-rates-from-communicable-neonatal-maternal-nutritional-diseases). DALY (disability adjusted life years).

While many of the initial restrictive measures have been lifted or relaxed in many contexts (although lockdowns have been and will likely continue to be reimposed depending on dynamics of COVID-19 transmission), their effects continue to ripple across time and space, particularly as they interact with baseline vulnerabilities which have also been exacerbated by the pandemic. Indeed, even before the pandemic, LMIC populations faced disproportionately high risks of communicable, neonatal, maternal and nutritional diseases which lead to early death (see figure 1), as well as rising rates of non-communicable diseases (NCDs).

These health risks have long been interlinked with poverty and economic precarity: large swathes of people in LMICs rely on informal livelihoods and already lacking social protection, have now also lost income earning opportunities due to the pandemic. The World Bank has estimated an additional 71 million people may be pushed into extreme poverty,^19^ severely compromising their ability to access health resources including safe and adequate housing, water, food and healthcare.

#### Vulnerable social groups

Just as countries are not equally vulnerable to broader health system and health impacts, people within countries, communities and households face different levels of risk. Marginalised social groups are likely to be impacted much more acutely by both supply-side and demand-side drivers described previously, as well as face sets of challenges that are unique to their own circumstances and context. Table 3 highlights additional challenges faced by particularly vulnerable groups. Categories may also often overlap, compounding vulnerability for people who live at their intersections.

**Table 3:** Social groups especially vulnerable to broader health impacts

Social group	Additional explanation
People Living with Disabilities (PLWD)	Restrictive measures compound difficulties already experienced by PLWD in accessing health services, especially in LMICs where 80% of PLWD reside.^63 64^ Many PLWD require homecare and personal assistance services, which are complicated by social distancing measures. During lockdown in India, women living with disabilities found themselves without this crucial support as their carers were stopped by police, or simply did not turn up.^65^ People with sensory or cognitive disabilities are also at risk of being excluded from crucial information, not only about COVID-19 but also other health risks and changes to service delivery if messaging is not disability-inclusive.
Elderly people	Geriatric services are a neglected health sector, and yet elderly are more likely to have pre-existing health conditions, and require/rely on support.^66^ Many—particularly women—may be also be caregivers themselves. Interruptions to care or ability to provide it, along with increased isolation, may increase risks for elders.^67^ Elder abuse rose in some countries such as Nepal, where police received nearly three times as many calls regarding the abuse of older people between March and May of 2020 as between December 2019 and February 2020.^68^
Women and girls	Entrenched gender discrimination puts women and girls at higher risk of malnutrition, poor sexual and reproductive health outcomes, and not receiving needed healthcare. Income losses and increased food insecurity may exacerbate these risks, especially if scarce resources are prioritised for men and boys. Isolation at home with abusers has also increased risk of SGBV. In Nigeria, available data showed a monthly increase of 149% in reports of gender-based violence following the introduction of lockdowns.^69^
Refugees and displaced people	Refugees and displaced people on the move may be less able to reach places of refuge amidst travel restrictions and tightened border controls.^70^ Those in crowded camp settings already facing greater health risks (including risk to COVID-19) may see reduced availability of services, including those for health and safety, if aid workers’ access to camps is restricted, as was reported in South Sudan.^71^ Refugees and displaced may also be at heightened risk of trafficking.^72^
Migrant workers	Millions of informal migrant workers lost their jobs due to the pandemic. On top of increased economic precarity, they faced lockdowns alone with little social support, and in countries such as India, millions struggled to return to their homes amidst suddenly imposed restrictions.^73^ International migrants may face heightened challenges as they may have no recourse to public assistance, may not speak local languages well, and may face stigma and blame for the virus. In June 2020, the International Organization for Migration estimated that a fifth of 6.3 million foreigners in Egypt were ‘vulnerable’ and in need of help.^74^
People in conflict-affected settings	War or prolonged unrest has left some health systems even less able to cope with additional stresses brought on by COVID-19.^75^ Further, conflict already impacts movement and socioeconomic and psychosocial well-being, and increases SGBV and recruitment into armed groups (including of children). Restrictive measures can worsen these conditions and risks, especially for women and children. Armed groups have also taken COVID-19 response into their own hands. In rural Colombia, they have violently suppressed and even killed people breaking restrictions.^76^
Racial, ethnic and religious minorities	Minority groups which already face discrimination and violence in their communities may face additional hurdles when seeking basic healthcare, including being outright denied treatment.^77^ Minorities may also be blamed for COVID-19, and correspondingly discriminated against. In Pakistan, a number of public authorities announced measures specifically targeting and restricting the movements of Hazara Shia, prior to any formal overall lockdown.^78^

PLWD, People living with disabilities; SGBV, sexual and gender-based violence.

## Documenting broader health system and health impacts

### Limited evidence and limited prioritisation

Research priorities and data collection have focused on COVID-19, with far less attention given to other health issues and impacts. Indeed, it is difficult to collect or monitor data in any fast-moving emergency, particularly in LMICs where robust data collection mechanisms are weak to non-existent. Broad modelled estimates, based on assumptions, past experiences and emergent understandings predominate, alongside qualitative descriptions in the media and from responding agencies and organisations. Although attention to broader health system and health impacts has been growing,^1^ it remains urgent to expand research, understanding and response in these areas.

One indicator of broader health impacts is the number of deaths in excess of expected mortality not attributed to COVID-19. However, quality data of this nature are limited in LMIC settings,^20^ and in many cases, only proxies are available for its estimation. In Jakarta, for instance, burial data suggest nine times as many excess deaths from non-COVID-19 causes occurred from March to May 2020 as COVID-19 deaths.^21^ It is likely that some (or indeed, many) were caused by COVID-19 and have simply been missed as such due to limited testing and surveillance capacities. Nevertheless, substantial additional mortality is clearly occurring,^22^ likely reflecting limited access or use of crucial healthcare.^23^ Both qualitative and quantitative investigations need to be deployed to better understand the extent, character and causes of impacts and to provide ‘actionable’ data.^24^

### Evidence of impacts

As earlier discussed, broader health system and health impacts are being driven by a range of dynamics. Some health services, such as child immunisations, antimalaria campaigns, HIV and TB screening and treatment, screening and treatment for NCDs and sexual and reproductive health services, have been particularly affected.

Table 4 offers an overview of impacts, including both modelled estimates and more selective evidence, in several health areas.

**Table 4:** Broader health system and health impacts in a range of health areas

Health area	Additional explanation
NCD	77% of 160 countries reported disruptions to NCD services, including rehabilitative services, hypertension, diabetes and asthma management, palliative care, dental care, cancer treatment and cardiovascular emergencies in a WHO assessment.^49^ Data and estimates from high-income settings reflected impacts on patients with cancer, resulting from delays in screenings, diagnosis and treatment, including reduced 5-year survival rates,^79^ and up to a 10% increase in cancer deaths over the next 5 years.^80^ While similar systematic modelling on NCD impacts in LMICs remains a major gap, individual examples have been documented. In India for instance, where 130 000 people rely on regular dialysis treatment for kidney conditions to stay alive, there were several reports of patient deaths due to dialysis equipment and centres being shut off, shut down or inaccessible for a range of reasons.^81^
Acute and chronic infectious disease	The processing of diagnostics for TB and HIV has been impacted by resources being diverted to COVID-19 response, and stockouts of antiretroviral drugs have been reported across 73 countries.^82^ In India, notifications of new TB diagnoses dropped 80% in May 2020.^83^ In June, 73% of 106 surveyed countries reported malaria service disruptions.^45^ Models taking service disruptions and reduced access (lack of screening, diagnosis and treatment) into account suggested sub-Saharan Africa, which bears 90% of the global malaria caseload, could witness a doubling of cases and up to 700 000 additional malaria deaths.^84^ An additional 500 000 AIDS-related deaths (including those due to TB) were also estimated.^85^ Globally, an additional 6 million cases of TB and 1.4 million deaths were predicted, setting back gains by 5–8 years.^86^ National programmes to eradicate neglected tropical diseases (NTDs) (eg, sleeping sickness and intestinal worms) which impact the world’s most marginalised were disrupted or suspended.^87^
Sexual, reproductive and newborn health	Although little context-specific evidence was yet available at the time of research, that which was, was alarming. In Nepal, institutional births reduced by over half during lockdown.^88^ Broader modelling had estimated a 39.3%–51.9% reduction in coverage for maternal health services over 6 months would result in 56 700 additional maternal deaths across 118 LMICs.^89^ Another study estimated an additional 28 000 maternal and 168 000 newborn deaths could result from just a 10% decline in relevant care coverage over a year, while 1.7 million women and 2.6 million newborns could suffer major complications across 132 LMICs.^90^ A similar estimated 10% decline in contraceptive use over a year was estimated to leave nearly 50 million women across 132 LMICs with unmet birth control needs and result in 15 million additional unintended pregnancies.^90^ Disruptions to abortion services were also estimated to lead to an additional 3.3 million women resorting to unsafe procedures.^90^ Sexual and gender-based violence has also risen considerably, with calls to support services increasing threefold in some settings.^46^
Children’s health	One study suggested 1.2 million additional children under 5 years old could die over 6 months across 118 LMICs, a 45% increase in child mortality, (assuming disruptions similar to what occurred during the West African Ebola epidemic).^89^ Increases in wasting due to malnutrition (which contributes substantially to under five deaths) may be a significant factor in this additional mortality.^91^ Globally, it was estimated an additional 10 million children could be pushed into acute malnutrition.^92^ Vaccine-preventable diseases are likely to be another major cause of child deaths. As of April 2020, 13.5 million children were thought to already have missed polio, measles, HPV, yellow fever, cholera and meningitis vaccinations,^93^ while there was concern that as many as 117 million would ultimately miss measles vaccinations alone.^94^ More granular research from Karachi found a 63%–90% decrease in routine immunisation visits during lockdown in slums and poor suburbs of the Pakistani city.^44^ Although visits resumed slowly following lockdown, children who missed their immunisations there and elsewhere may ultimately never get them or may get them too late. Some countries, such as the Democratic Republic of Congo, were already reeling from outbreaks of vaccine-preventable diseases prior to the COVID-19 pandemic.^95 96^

LMICs, low-income and middle-income countries; NCD, non-communicable disease; TB, tuberculosis.

## Mitigating negative health impacts: health system and community approaches

COVID-19 must be put in perspective vis-à-vis other disease burdens and health services, including medium-term and long-term views. Prioritisation of health system resources should be set via meaningful participation of affected communities, health system users and patients, with special consideration for the participation of vulnerable groups. This requires funding and support for dedicated spaces and staff to bring the public (including community members, civil society organisations and grassroots movements) and policy makers together for inclusive dialogue.^25^ Past experiences from the HIV and West African Ebola epidemics demonstrated how community involvement was crucial to improving response.^25^ Coordination between international, national and local actors also proved critical to raising awareness and prompt action. Tools including evidence-to-decision frameworks and systematic trade-off appraisal can also be leveraged to support better informed short-term decision-making.^26 27^

COVID-19 has shed light on health system capacity and the importance of preparing for and addressing comorbidity. Emerging concerns about other infectious conditions (measles, TB and HIV/AIDS) and NCDs in their relation to COVID-19 create opportunities to incorporate these priorities into a more integrated, health system approach. A positive experience drawn from the fight against HIV/AIDS is the importance of tracking the disease and its treatment and holding governments accountable for containing the spread and ensuring universal treatment coverage.^28^ The global community and LMICs should be accountable to the commitments to primary healthcare made at Alma Ata and to Universal Health Coverage commitments, and donor countries should provide necessary funding and support. This global support for health system strengthening in LMICs is also crucial to avoid a medium-term scenario in which COVID-19 is addressed in wealthier countries, but remains endemic in poorer ones, as has occurred with diseases such as cholera.^29^

In the wake of COVID-19, NCD response, locally relevant contagious disease and health services (eg, nutrition and maternal and child health), and their integration into universal and affordable primary healthcare should be a priority.^30^ The COVID-19 response and services should be integrated, whenever possible, within existing health and social programmes.^31^ These programmes could share information systems, infrastructures, diagnostic and treatment capacities and outreach to break siloes.^32^ Health systems should establish cross-sectoral links (eg to social protection and education) to incorporate the social determinants of health.^30^ Digital solutions—where feasible—can help build linkages across and beyond health systems.

Decentralised, community-based, and people-led approaches are more likely to reach people suffering from broader health impacts and be accepted by communities.^33^ Examples of community-based approaches have been implemented to address COVID-19-associated service disruption in some settings. In India, for instance, volunteer health workers shared tablets and phones, and went door-to-door (while maintaining physical distance) to find cases, deliver antiretroviral therapy, ensure treatment uptake, deliver food, and give advice on HIV and COVID-19 transmission.^34^ In sub-Saharan Africa, community health workers have similarly delivered bed nets, medications and supported people to sustain needed treatment.^35^ The lack of personal protective equipment (PPE), however, has been an important challenge. Investing in financial resources and capacity building for community workers is crucial to prevent future outbreaks.^30^

Health provision for COVID-19 and its broader health system and health impacts must build on existing response networks: civil society organisations (unions, professional associations, religious groups and women’s groups) and social movements (within and beyond health). These organisations and movements should be provided with resources and support to lead elements of response and healthcare delivery. In turn, health policy makers should consider, wherever relevant, the plurality of health providers and therefore engage with private clinicians, pharmacists, drug sellers, traditional and faith healers, herbalists and others who may be patients’ first point of healthcare. These providers should be awarded necessary resources and skills for infection prevention (including PPE), in order to support triaging, surveillance, diagnosis and treatment for a range of health issues.

Real-time surveillance of perceptions, delivery, access to and use of health services can enable policy makers and responders to take immediate, context-relevant action.^36^ Expanded collection and use of granular social science research should be put into place to identify what health services are disrupted and why, to understand localised impacts, and to guide local and national response action. A framework for integrated data analysis, such as the integrated, multisectoral outbreak analytics (IMOA) model can provide a comprehensive understanding of cause and effect of broader impacts at multiple levels. IMOA brings together data on behaviour, perceptions, health service use, epidemiological trends of other health outcomes, movement mapping and market prices against a timeline of applied NPIs.^37^

These systems would also support accountability of local-level health providers and policy makers through public monitoring of health indicators and services, and integration of patient and community feedback to improve health services. Over time and integrated alongside a diversity of natural and social science data considered through multisectoral deliberative processes,^36^ this surveillance can also feed into joined-up medium-term and longer-term approaches and commitments up to the task of addressing the triple threat of COVID-19, other serious health priorities and impending economic crises into the future.

## Conclusion

In just a few months, COVID-19 fundamentally changed the ways in which our social, economic and political systems operate. Not least among these are our health systems. While some changes have been necessary (to prevent in-person health service visits from becoming sites of COVID-19 infection and to allocate scarce resources), they have not necessarily been well conceived or executed, in part due to a lack of resources. Furthermore, the fallout from the interactions between vertical response measures, pre-existing vulnerabilities and wider impacts of the pandemic have led to myriad new challenges and barriers for health systems and for people who need care. The lack of attention to the broader health system and health impacts of vertical response measures, particularly under lockdowns—but also other elements of public health responses which, while less directly disruptive than lockdowns, may still have blind spots—has resulted in and will continue to cause significant harm to health and well-being. LMICs are particularly vulnerable as their substantial disease burdens and historically weak health systems present significant challenges. In addition to documenting some of the pathways and evidence of these impacts, this paper calls for more holistic approaches to health in the context of COVID-19, but also beyond it, and urges action to mitigate tragedy in both the short- and long-term.
