# Supplementary material for: COVID-19 response: mitigating negative impacts on other areas of health
Source: BMJ Glob Health. 2021 Apr 15;6(4):e004110. doi: 10.1136/bmjgh-2020-004110 (PMC8053814; doi:10.1136/bmjgh-2020-004110)
Supplement: Supplementary data [file bmjgh-2020-004110supp001.pdf]

APPENDIX

This paper is a version of a longer report prepared for the Social Science in Humanitarian Action Platform (SSHAP), a project which aims to provide rapid evidence syntheses and analyses to operational actors engaged in humanitarian response to help them respond more effectively. Given urgency and the emergent nature of our topic, we engaged a 'rapid evidence assessment' methodology to summarise and synthesise findings (Nobel and Smith 2018: https://ebn.bmj.com/content/21/2/39).

We began our assessment by searching the academic database Web of Science using combinations of the search terms: ("secondary" or "collateral" or "indirect") and "health" and "impacts" and ("COVID-19" or "coronavirus" or "pandemic"). The search year was restricted to 2020, and we conducted the search in August 2020. The search yielded very few relevant articles, likely reflecting the limited peer reviewed literature available at that point, and due to our wide scope which focused on broader health and health system impacts rather than on any particular health or health system issue, context, pandemic response, or impact for which we could employ a more targeted search.

We extended our search to more general Google and Google scholar searches, as well as invited colleagues to share papers and reports that spoke to our query. In addition to academic material, including published articles, pre-prints, reports and blogs, we drew extensively also on grey literature identified through our searches. This included reporting from news media outlets, as well as development, public health and humanitarian organisations, including material identified through the humanitarian database ReliefWeb, and a resource inventory being maintained by the Center for Global Development on the indirect health effects of the pandemic which included both academic and grey sources (https://docs.google.com/spreadsheets/d/1_MBh3FmuSZK-9UQsrBYaYtIHdCqnez9oqJ8t216LkbE/edit#gid=484814669). We also engaged extensive snowballing techniques and reference mining, and drew on the experience of author SC who is directly engaged in pandemic response. This iterative strategy enabled us to include the most recent developments, and capture data not represented in formal published research in a fast changing and emergent situation.

While we aimed to be as comprehensive as possible under the time constraints and circumstances of a fast-changing situation, we are likely to have missed relevant studies and reporting. Furthermore, as our scope was very broad, we were unable to cover every relevant issue and area.
